# Increased T cell breadth and antibody response elicited in prime-boost regimen by viral vector encoded homologous SIV Gag/Env in outbred CD1 mice

**DOI:** 10.1186/s12967-016-1102-7

**Published:** 2016-12-20

**Authors:** Anne-Marie Carola Andersson, Peter Johannes Holst

**Affiliations:** Department of Immunology and Microbiology, Center for Medical Parasitology, University of Copenhagen, Copenhagen, Denmark

**Keywords:** Adenoviral vectors, Human immunodeficiency virus, Vaccine, Virus-like particles

## Abstract

**Background:**

A major obstacle for the development of HIV vaccines is the virus’ worldwide sequence diversity. Nevertheless, the presence of T cell epitopes within conserved regions of the virus’ structural Gag protein and conserved structures in the envelope (env) sequence raises the possibility that cross-reactive responses may be induced by vaccination. In this study, the aim was to investigate the importance of antigenic match on immunodominance and breadth of obtainable T cell responses.

**Methods:**

Outbred CD1 mice were immunized with either heterologous (SIVmac239 and HIV-1 clade B consensus) or homologous (SIVmac239) gag sequences using adenovirus (Ad5) and MVA vectors. Env (SIVmac239) was co-encoded in the vectors to study the induction of antibodies, which is a primary target of current HIV vaccine designs. All three vaccines were designed as virus-encoded virus-like particle vaccines. Antibody responses were analysed by ELISA, avidity ELISA, and neutralization assay. T cell responses were determined by intracellular cytokine staining of splenocytes.

**Results:**

The homologous Env/Gag prime-boost regimen induced higher Env binding antibodies, and induced stronger and broader Gag specific CD8+ T cell responses than the homologous Env/heterologous Gag prime-boost regimen. Homologous Env/heterologous Gag immunization resulted in selective boosting of Env specific CD8+ T cell responses and consequently a paradoxical decreased recognition of variant sequences including conserved elements of p24 Gag.

**Conclusions:**

These results contrast with related studies using Env or Gag as the sole antigen and suggest that prime-boost immunizations based on homologous SIVmac239 Gag inserts is an efficient component of genetic VLP vaccines—both for induction of potent antibody responses and cross-reactive CD8+ T cell responses.

## Background

The ideal human immunodeficiency virus (HIV) vaccine should likely activate both the humoral and the cellular arm of the immune system; enabling antibodies to neutralize incoming virus particles while CD8+ T cells would kill already infected cells and limit the spread of the virus.

The substantial role of CD8+ T cells in the control of HIV type 1 (HIV-1) infection is supported by the correlate of protection with specific HLA class I allotypes [1–4], a temporary decline in peak viremia coinciding with appearance of CD8+ T cell responses during acute infection [5, 6], and occurrence of escape mutants in response to specific CD8+ T cells [7, 8]. In humans, protective CD8+ T cell responses have been found to correlate with the targeting of Gag [9, 10] and possibly of Nef [11], while inversely correlating with responses against envelope (Env) [10]. These correlations have been substantiated by additional population studies that have generated in-depth data by carefully screening responses to the HIV-1 proteome and investigating protective responses towards individual epitopes [12–17]. Together, these studies indicate that it is theoretically possible to successfully target HIV-1 by inducing T cells targeting specifically effective epitopes. However, such protective T cell specificities have only been raised infrequently after immunization with full length HIV-1 antigens [18] and previous vaccination attempts that aimed to induce CD8+ T cells have failed to control HIV-1 replication overall [19]. Despite their overall clinical failure, these studies did provide evidence of improved virus control after polyspecific targeting of defined regions of Gag, Vif, Nef, and Pol [18, 20]. A shift to more focused constructs aimed to direct CD8+ T cells specifically to carefully selected regions has therefore been a logical step forward. Conserved regions vaccines [14, 21] and conserved elements (CE) vaccines [22] each represents approaches aiming to overcome the challenge of HIV-1 diversity and raise responses to rarely targeted, yet protective specificities [23].

While T cell based vaccines have yet to show efficacy in clinical trials, the RV144 trial demonstrated the possibility of reducing infection risk by raising Env specific antibodies with a virus-like particle (VLP) encoded virus vectored prime and a protein boost vaccine [24]. A recent interest in the coordinated responses against Gag and Env has emerged. Studies by Schell et al. suggested that Gag benefits a response based on protective antibodies in a vaccine secreting VLPs [25, 26]. Additionally, the immune response against Gag has been shown to support antibody responses against Env displayed on VLPs by providing an additional source of intrastructural T cell help [27, 28].

Due to the additive and potentially synergistic value of using Gag and Env for effective antibody and T cell mediated protection, we sought to study the induction of potentially protective Gag and Env responses in VLP based virus vectored immunization regimens. To investigate the possibility of inducing T cell responses against conserved or cross-reactive epitopes in the Gag polyprotein, while preserving antibody responses towards Env trimers in such a virus vectored VLP vaccine construct, we used a prime-boost regimen with heterologous Gag together with homologous Env and compared it to a homologous Gag/Env regimen. We did this by utilizing a combination of adenoviral vectors co-encoding SIVmac239 *env* and either HIV-1 or SIVmac239 *gag* sequences as primers for modified vaccinia Ankara (MVA) vectors encoding SIVmac239 *env* and SIVmac239 *gag*. We included a non HIV sequence as previous studies have found no benefit of prime-boost regimens using Gag from different HIV clades while improved breadth was found using different HIV Env clade sequences [29, 30]. Using SIV and HIV Env provides a diversity resembling the successful heterologous Env sequences and results in a vaccine design immediately testable in a non-human primate model. Here we show that outbred CD1 mice immunized with homologous Gag/Env had an increased breadth and functionality of T cell responses against heterologous *gag* sequences as well as a surprising increased magnitude of Env specific antibody responses. Mice immunized with homologous Gag selectively expanded Gag specific T cells following the booster immunization whereas mice immunized with heterologous Gag selectively expanded Env specific T cells following the boost. These data highlights the importance in the selection of *gag* sequences in VLP encoding virus vectored immunization regimens.

## Methods

### Mice

Female CD1 mice at the age of 6–8 weeks were obtained from Scanbur (Denmark). The mice were allowed to acclimatize for one week prior to the initiation of an experiment. All experiments were performed according to national guidelines and experimental protocols approved by the national animal experiments inspectorate (Dyreforsøgstilsynet).

### Adenoviral vaccine production

HIV-1 clade B consensus (HIV-1 CON B) or SIVmac239 *gag* was encoded after a CMV promoter, followed by a self-cleavable P2A peptide and then by SIVmac239 *env* and SV40 polyA. The expression cassette was cloned into a human adenovirus type 5 backbone and produced, purified and titered as described [31]. A modified vaccinia Ankara (MVA) vaccine encoding SIVmac239 *gag*, *pol*, *env* (truncated at aa 733) (MVAgpe) [32] was kindly provided by Dr. Patricia Earl (Laboratory of Viral Diseases, NIH). The vaccine was amplified and titered in primary chicken embryoblasts according to the protocols in Kramer et al. [33].

### Purification of vaccine encoded VLPs for further characterization

Vero cells were infected with 50 plaque forming units (PFU)/cell of either Ad5 vaccine, supernatants harvested 48 h post infection, and VLPs concentrated as previously described [31]. Pellets were resuspended in PBS at 280 X of the original concentration.

### Western blot analysis

VLPs purified from Ad5 infected vero cells were prepared as previously described [31] and characterized by western blot. Env was detected with the SIVmac251 gp120 specific monoclonal antibody (mAb) KK46 [34] [NIH AIDS Research and Reference Reagent Program (NARRRP)] followed by HRP coupled goat anti-mouse immunoglobulin antibody (Dako). The same blot was analysed for the presence of Gag using HIV-1 anti-p24 mAb 183-H12-5C [35] (NARRRP), and goat anti-mouse immunoglobulin antibody (Dako). SIVmac239 gp130 [36] and SIVmac251 BK28 pr55 Gag were loaded as positive controls (NARRRP). The blots were developed using ChemiLucent Detection System kit (Pierce). Analysis was performed using Image Studio Lite software (LI-COR Biosciences).

### Cell surface expression analysis

Env expression was analysed on the surface of vero cells 2 days after infection with 50 PFU/cell of either Ad5 vaccine. Cells were stained with the ITS52, ITS03, and ITS40 mAbs [37] (kindly provided by Dr. Mario Roederer, VRC, NIAID, NIH). Binding of the mAbs was detected using anti-human IgG Fc-APC antibody (BioLegend), and the cells were acquired using an LSRII instrument (BD Biosciences) and analysed with FlowJo software (Tree Star, Ashland, OR).

### Immunizations

Groups of mice (5 or 10 per group) were immunized intramuscularly (i.m.) with 2 × 10^8^ IFU with either Ad5 vaccine. Where indicated, mice were boosted i.m. 59 days after priming with 1 × 10^7^ IFU of MVAgpe. Vaccines were applied in a total volume of 50 μl PBS in the quadriceps muscle, changing legs at each immunization time-point.

### Antibody response measurements

Antibody responses against SIVmac239 Env were determined in a concanavalin A (ConA) (Sigma-Aldrich) enzyme linked immunosorbent assay (ELISA) as adapted from [38]. SIVmac239 Env was produced in 293FT cells by co-transfection of a shuttle plasmid encoding SIVmac239 Env and the plasmid pSG3^Δenv^ [39] (NARRRP). ELISA was performed as described elsewhere [31]. Avidity measurements were performed simultaneously with antibody response measurements as adapted from [38], using 0.1 M sodium citrate buffer (pH 3.0). The avidity index was calculated by dividing the titer obtained with sodium citrate treatment by the titer obtained without sodium citrate treatment, and multiplied by 100.

### SIV Env pseudovirus production and titration

The Env constructs for SIVmac239 and SIVsmE660.11 pseudovirus production were obtained from Dennis Burton (The Scripps Research Institute) and David C. Montefiori (Duke University), respectively. Production and determining TCID has been described elsewhere [40].

### Neutralization assay

Neutralization of pseudoviruses was measured in TZM-bl cells following the protocols in Montefiori [40]. To account for unspecific binding to either pseudovirus, pooled pre-immunization serum samples were analysed at a dilution of 1:20. SIVmac251 antiserum [41] (NARRRP) was included as a control. Neutralization against SIVmac239 was assayed in pools of 4–5 mice per group. The SIVmac239 pseudovirus did not achieve the RLU of 10 times the background (5.2 times the RLU of the background), but was included in the analysis since it was clear that there was no neutralization in neither sample that was tested.

### Intracellular cytokine staining

Intracellular staining was performed on splenocytes using a standard protocol [42] using 0.67 μg/ml HIV-1 CON B Gag pool, SIVmac239 Gag pool, SIVagm vervet Gag pool, SIVmac239 Env pool, CE pools for the three different Gags respectively) (all from NARRRP) at 37 °C and 5% CO_2_. Based on definitions by [22], CE pools were prepared from peptides spanning the CE from whole Gag peptide sets. The following antibodies were used for detection (Biolegend): CD8_PerCP.Cy5.5, CD4_FITC, B220_Pacific Blue, CD44_APC.Cy7, IFN-γ_APC, TNF-α_Pe.Cy7. The cells were acquired using an LSRII instrument (BD Biosciences) and analysed with FlowJo software (Tree Star, Ashland, OR). One mouse was excluded from the T cell analysis due to the collection of too few events.

### Statistical analysis

Nonparametric Mann–Whitney tests were performed for analysis of differences between the groups. Spearman rank testing followed by Holms correction for multiple comparisons was used to assess correlations. The ID50 values in the neutralization assay were calculated in Graph Pad Prism using an asymmetric curve fitting (5 parameters). Statistical analyses were performed using either the R statistical software or Graph Pad Prism. p values <0.05 were considered significant.

## Results

### Vaccine design and characterization

**Fig. 1:**
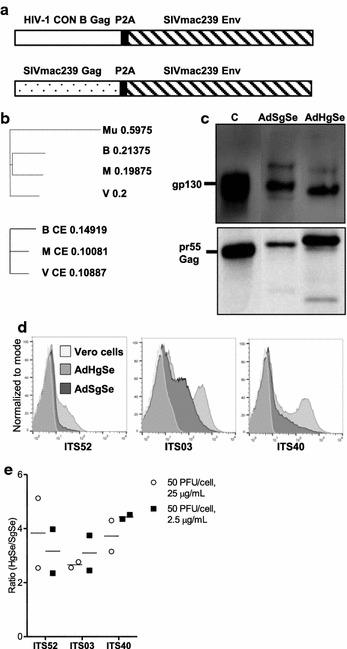
Vaccine design and vaccine characterization. **a** Schematic representation of Ad5 vectors encoding either SIVmac239 *gag* or HIV-1 CON B *gag*, P2A preceded by a glycine-serine-glycine linker (not noted in the figure), and SIVmac239 *env*. The inserted antigens were flanked by huCMV and a SV40 polyadenylation signal. **b** Phylogenetic tree of Gag used in the Ad5 vaccines and for analysis. The distance values show the number of substitutions as a proportion of the length of the alignment (here excluding gaps). *Upper panel* is analysis of Gag and lower panel is analysis of p24 CE. *Mu* murine leukemia virus, used as an outlier; *H* HIV-1 CON B Gag; *M* SIVmac239 Gag; *V* SIVagm vervet Gag. **c** Gp160 and gp120 were detected in ultra centrifuge purified VLPs from the supernatants of Ad5 infected vero cells by SDS-PAGE (reduced) followed by western blot. SIVmac251 specific mAb KK46 was used for detection of gp120. Pr55 Gag and pr55 Gag cleavage products were detected using 183-5C-H12 HIV-1 p24 specific mAb. SIVmac239 gp130 and SIVmac251 BK28 pr55 Gag served as positive controls for gp120 and pr55 respectively. **c** is control lane. **d** Surface staining of vero cells infected with Ad5 vaccines (50 PFU/cell, 25 µg/ml mAb) or uninfected with indicated mAbs. **e** Ratio of AdHgSe to AdSgSe mAb binding calculated as mean fluorescence intensity × positively gated fraction. Calculations were performed on cells infected with 50 PFU/cell of either vaccine as in **d**, using 25 or 2.5 µg/ml mAb concentration. Shown are calculated ratios from two independent assays with the mean marked by *horizontal lines*

To enable shift of the *gag* sequence in otherwise identical viral vectored prime-boost regimens, we encoded SIVmac239 *env* with either a SIVmac239 *gag* sequence (AdSgSe), or HIV-1 CON B *gag* sequence (AdHgSe) in individual Ad5 encoded VLP expression cassettes for use as priming vaccines (Fig. 1a). The adenovirus vectors were used as priming immunogens for MVA vectors encoding SIVmac239 *gag*, *pol* and *env* (MVAgpe) likewise designed to secrete VLPs [32]. T cell immune responses were analysed to vaccine incorporated *gag* sequences and a more distantly related *gag* sequence (SIVagm vervet). We chose the SIVmac239 *gag* sequence and the HIV-1 CON B *gag* sequence to study heterologous Gag immunization in virus-vectored VLPs in a vaccine design suitable for nonhuman primate testing. SIVagm vervet was chosen to measure cross-reactive responses as it had the same sequence similarity to either HIV-1 CON B *gag* or SIVmac239 *gag*, and similar similarity to these as the similarity between HIV-1 CON B and SIVmac239 *gag* (illustrated in Fig. 1b). The two Ad5 vaccines were characterized for VLP secretion of the respective vaccine antigens in the supernatant of vaccine infected vero cells. VLPs were collected and Gag and Env content was assessed by western blotting (Fig. 1c). We detected the expression of Gag with the HIV-1 p24 mAb 183-H12-5C, previously shown to cross-react with SIVmac239 Gag [43]. Bands for both pr55 and pr55 cleavage products were observed for both constructs. For Env, both gp120 and gp160 bands were visible by probing with the gp120 specific mAb KK46. The MVAgpe vaccine had been characterized previously and this analysis was not repeated here except for verification of Env expression by immunocytochemistry (not shown and [32, 44]).

Surface staining of vero cells confirmed the expression of Env from both Ad5 vaccines. An increase in cell surface binding for the three antibodies utilized for analysis (ITS52, ITS03, ITS40) was observed for the vaccine AdHgSe compared to the AdSgSe vaccine (Fig. 1d). Both vaccines exhibited lower binding to the V3 specific mAb ITS52 as compared to the V2 specific mAbs ITS03 and ITS40, but except for the quantitative difference in cell surface binding, the two vaccines stained similarly with each antibody indicating similar presentation of the conformational epitopes recognized by these antibodies [37], (Fig. 1e).

### Heterologous and homologous Gag prime-boost regimen

**Fig. 2:**
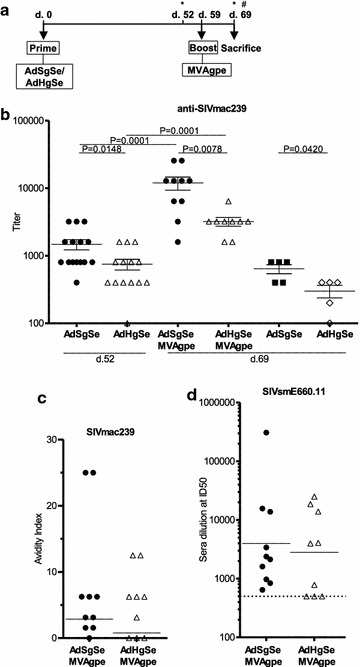
The magnitude of SIVmac239 Env responses is dependent on the co-encoded *gag* sequence. CD1 mice were primed with Ad5 vaccines co-encoding SIVmac239 *env* with either SIVmac239 *gag* (AdSgSe) or HIV-1 CON B *gag* (AdHgSe) as illustrated in the experimental outline (**a**). *Asterisk* indicates time of serum harvest. (#) indicates harvest of spleens. 10 respectively 9 mice from each group were then boosted with MVA encoding SIVmac239 *gag*, *pol*, and *env* (MVAgpe) truncated at amino acid 733. Immunogenicity was investigated 52 and 69 days post priming immunization and also 10 days post boosting with MVA (d.69). **b** Co-encoding SIVmac239 *gag* compared to HIV-1 CON B *gag* with SIVmac239 *env* led to significantly increased end point titers against SIVmac239 Env, both after priming immunization, and also after the MVA boost. **c** The Avidity Index was determined in the sodium citrate displacement assay against SIVmac239 Env. **d** Neutralization titers against SIVsmE660.11 were determined at serum dilution starting at 1:500 (indicated with dotted line). **b**–**d** depicts individual mice with horizontal lines indicating mean with SEM (**b**) or geometric mean (**c**, **d**)

The use of the two adenovirus vectors, AdHgSe and AdSgSe, and the MVAgpe vector allowed us to prime with either HIV-1 CON B Gag or SIVmac239 Gag followed by boost with SIVmac239 Gag, while keeping the SIVmac239 Env constant in the prime-boost regimen. Blood samples for antibody analysis were taken a week prior to boosting immunization and also 10 days post boost, at the same time as the spleens were harvested for T cell analysis (Fig. 2a), as this was anticipated as the time of maximal T cell responses [45] and peak antibody responses following an MVA booster immunization [46].

### Humoral responses

Env responses against VLP derived SIVmac239 Env were analysed 52 days post 1st immunization (Ad5) and 10 days post (d. 69) the second immunization (MVA). Differences in immune responses were observed between the two Ad5 priming regimens both after the first immunization and after the MVAgpe boost (Fig. 2b). Mice primed with AdSgSe had significantly higher end point titers against pseudovirus derived Env at both time-points (p < 0.05 and <0.01, respectively). Boosting with MVAgpe increased the mean responses eightfold for the AdSgSe primed group, and fourfold for the AdHgSe primed group. Mice that had only been primed with either Ad5 vaccine showed approximately a twofold decrease in Env responses after 69 days as compared to 52 days post immunization. Still, at this time-point, 69 days post immunization; titers remained significantly higher for the group immunized with AdSgSe as compared to AdHgSe (p < 0.05).

The immunoglobulin avidity was also determined in the two groups of mice that had received boosting immunizations with no observed differences in the immunoglobulin avidity index between the immunization regimens either before or after the boost (data only shown after boost) (Fig. 2c).

Neutralizing responses were determined against SIVsmE660.11, categorized as a neutralization sensitive tier 1 [47]. The ID50 titers were analysed for the two groups of mice that had been boosted with MVAgpe. Mice primed with AdSgSe, all had detectable ID50 titers, while the ID50 titers of 3 mice in the AdHgSe primed group were below the chosen start dilution of 1:500 for detection (Fig. 2d).The geometric mean titers were similar; hence, no significant differences were detected. Neutralization was also analysed in pools of 4–5 mice against the homologous SIVmac239, classified as a neutralization resistant tier 3, with no neutralizing responses detected (data not shown).

### T cell responses to homologous and distantly related Gag sequences

**Fig. 3:**
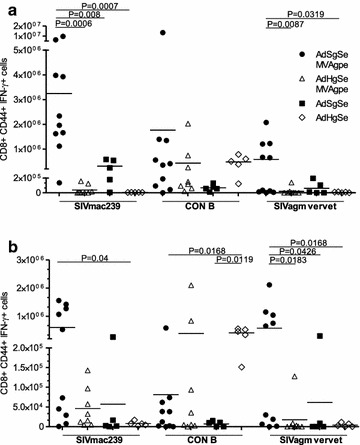
Heterologous Gag immunization does not induce more cross reactive T cell responses. At day 69 mice were sacrificed and spleens were harvested for analysis of vaccine induced T cell responses against HIV-1 CON B Gag, SIVmac239 Gag, and SIVagm vervet Gag. **a** Number of IFN-γ positive CD8+ T cells measured at day 69 against full length Gag. **b** Number of IFN-γ positive CD8+ T cells measured at day 69 against CE of the three Gag variants. Shown are calculated total numbers of responding CD8+ T cells per spleen of individual mice. *Horizontal lines* depict the mean of the groups

The frequency and epitope specificity of T cell responses were analysed by intracellular cytokine staining on splenocytes 10 days after the MVA boost (69 days post prime). Responses were analysed against the vaccine antigens, and against SIVagm vervet Gag (Fig. 1b), to reveal the breadth and/or the cross-specificity of the responses. The homologous boost was found to increase mean responses towards all 3 complete Gag peptide pools, whereas the heterologous boost induced HIV-1 CON B biased responses that were not increased after the boost (Fig. 3a). Also, quite prominently, the homologous Gag immunization induced significantly higher CD8 + IFN-γ responses against SIVagm vervet Gag. Examination of T cell responses to CE within p24 of Gag revealed that heterologous Gag immunization was not beneficial for the induction of conserved p24 responses either (Fig. 3b). Instead, homologous Gag immunization induced significantly higher CD8 + IFN-γ responses against SIVagm vervet CE, with similar responses towards the CE in HIV-1 CON B Gag. A generally higher magnitude of responses against SIVmac239 Gag CE was also observed in the homologous vaccination group compared to the heterologous vaccination group, and prominent responders was restricted to this group (5/10), but this difference was not significant using non-parametric statistics.

**Fig. 4:**
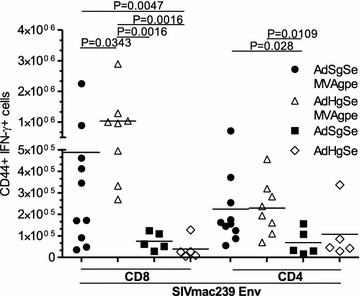
Heterologous Gag immunization induces high numbers of Env specific CD8+ T cells. Mice were analysed for respective CD4+ and CD8+ T cell responses at day 69 upon sacrifice and harvest of spleens for analysis. Frequency of IFN-γ positive CD8+ and CD4+ T cells against SIVmac239 Env. Shown are calculated total numbers of responding CD8+ T cell per spleen of individual mice. *Horizontal lines* depict the mean of the groups

Analysis of the frequency of T cell responses against SIVmac239 Env showed that heterologous Gag immunization led to significantly higher CD8 + IFN-γ responses against SIVmac239 Env (p < 0.05) (Fig. 4). The MVA immunization increased the responses by 30-fold in this group as compared to homologous Gag where the responses were insignificantly expanded, and only by approximately fourfold. The CD4+ T cell responses against Env in these two groups were very similar (Fig. 4).

**Fig. 5:**
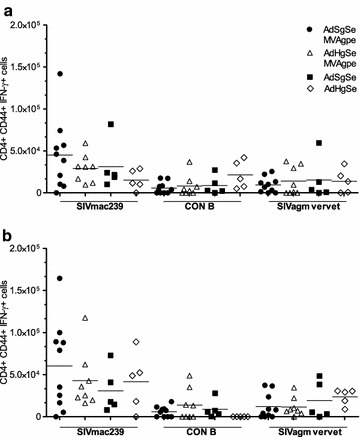
Frequency of CD4+ T cells against Gag and CE. **a** Splenocytes were harvested at day 69 and CD4+ T cells were analysed for their responses against Gag variants (**a**) and CE variants (**b**). Shown are calculated total numbers of responding CD4+ T cell per spleen of individual mice. *Horizontal lines* depict the mean of the groups

CD4 + T cell responses were also determined against whole protein of Gag variants and CE variants revealing generally low numbers of CD4 + IFN-γ responses against variants of Gag and CE (Fig. 5a, b).

### Correlations between different T cell specificities and between T cells and antibody responses

Spearman rank correlations were determined within the groups for the measured Gag specific CD8+ T cell specificities and the multiple comparisons were corrected using Holm’s correction. Possible synergistic T and B cell responses were also addressed.

**Fig. 6:**
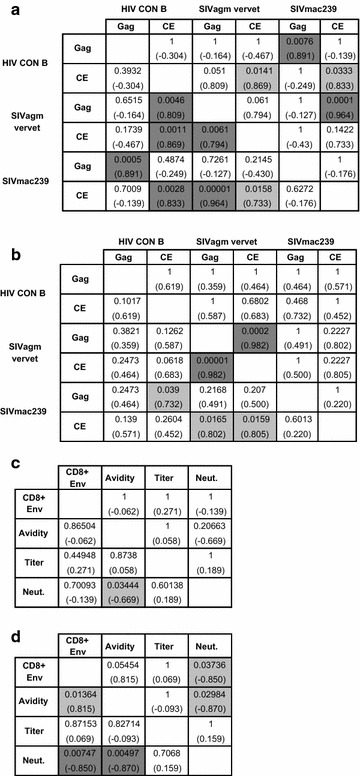
Correlation data of CD8 + IFN-γ responses and humoral responses. **a** Correlations between CD8 + IFN-γ responses towards the different Gag and CE in the homologously boosted mice. **b** Correlations between CD8 + IFN-γ responses towards the different Gag and CE in the heterologously boosted mice. **c** Correlations between CD8 + IFN-γ responses and humoral responses towards Env in the homologously boosted mice. **d** Correlations between CD8 + IFN-γ responses and humoral responses towards Env in the heterologously boosted mice. Exact p values are written within the cells with correlation coefficients shown in *parenthesis*. The *lower left* depicts uncorrected p values while the *upper right* depicts p values obtained after Holms correction for multiple comparisons*. Dark gray* shadow refers to p < 0.01, light grey shadow refers to p < 0.05. CD8+ Env is CD8 + IFN-γ responses against Env. Titer is end point titers at day 69. Neut is neutralizing responses at day 69

In homologous Gag immunization, CD8 + IFN-γ responses against SIVagm vervet CE correlated with HIV-1 CON B CE CD8 + IFN-γ responses (p < 0.05) (Fig. 6a). Similarly, CD8 + IFN-γ responses against SIVmac239 CD8 + IFN-γ CE responses correlated with HIV-1 CON B CE CD8 + IFN-γ responses (p < 0.05), but not significantly with SIVagm vervet CE responses following correction for multiple comparisons.

In heterologous immunization, CD8 + IFN-γ responses against SIVagm vervet Gag correlated with its CE response (Fig. 6b).

Notably, the heterologous vaccination regimen showed a negative correlation between CD8 + IFN-γ T cell responses against Env to neutralizing antibody responses against SIVsmE.660.11 (p < 0.05), but a positive correlation trend with the avidity of anti-Env immunoglobulins (p = 0.05454) (Fig. 6d). No correlations between CD8 + IFN-γ responses against Env and humoral responses were observed for the homologous vaccination regimen (Fig. 6c).

## Discussion

The effect of the VLP scaffold Gag on the strength of virus encoded VLP vaccine induced T cell and antibody response has not been investigated previously, nor has the effect of using different *gag* sequences in heterologous prime-boost VLP encoding vaccines. In our analysis of antibody responses we found significantly higher Env immunoglobulin titers induced by the homologous Gag prime-boost regimen. In contrast, the two different immunization regimens induced similar levels of neutralizing antibodies against the neutralization sensitive tier 1 SIVsmE660.11, and also similar avidity of anti-Env antibodies. More interesting, in addition to higher Env binding titers, the analysis of T cell responses revealed a superiority of the homologous Gag regimen; also at inducing broader CD8+ T cell responses against Gag. Interestingly, and perhaps retrospectively explainable, it appeared that while Gag showed expected CD8+ T cell immunodominance over Env, homologous Env showed immunodominance over heterologous Gag in a prime-boost regimen.

Our investigation of conserved T cell responses and the selection of CE peptide pools for analysis has its foundation in studies by Kulkarni et al. [22, 23, 48], who have designed an antigen combining only conserved parts of p24 of Gag that normally correlates with increased virus control, and are found to include epitopes that cannot be mutated without virus fitness loss. Our homologous full-length Gag prime-boost regimen increased both the magnitude and breadth to full length Gag variants and CE, with the induced CE responses generally correlating with the overall responses to different virus variants. Bimodal responses were observed in particular for CE for both CD4+ and CD8+ T cells, reflecting the harbouring of different MHC alleles in CD1 mice. Heterologous Gag immunization did induce some CE responses, albeit not the cross-reactive responses that we had expected, reflecting the preferential Env specific T cell expansion in this group that appeared to come at the expense of negligible boosting of Gag specific responses. Compared to our results, Kaufman et al. reported an intermediate effect where heterologous full length insert in a prime-boost regimen neither increased, nor decreased the responses against conserved epitopes of Gag, but this included the weakly immunogenic Ad35 vector in the prime-boost regimen and no *env* was encoded [29]. The inclusion of Env in our prime-boost vaccinations likely makes a profound difference as the MVA immunization only boosted homologous CD8+ T cell responses in our study, and it would appear that homologous responses must have blocked expansion of potentially cross-reactive T cells recognizing the heterologous boosted antigens. A mechanism for how such preferential boosting of homologous antigens can increase breadth, as compared to a heterologous boost where conserved sequence specific T cells are preferentially re-stimulated, may have been provided by Kelly et al. [49] who found dominant and highly immunogenic epitopes to be effective at selecting for cross-reactive T cell responders, and Varela-Rohena et al. [50] who observed increased cross-reactivity of T cell receptors mutated for increasing affinity.

With regards to antibodies, both of the prime-boost immunization regimens analysed here had increasing antibody titers after being boosted. The priming vaccine encoding HIV-1 CON B *gag* was inferior in priming Env responses, having significantly lower antibody titers 7 weeks after priming and also 10 weeks post priming. These results implicate the benefit of SIVmac239 Gag as a VLP scaffold for induction of a binding antibody, although we could not observe any clear differences in the VLPs or transduced cells likely to explain this benefit. We also observed a fourfold difference in the geometric mean titers between the boosted groups; however, both the avidity of the anti-Env responses and neutralizing responses against SIVsmE660.11 were roughly similar.

An intriguing aspect of our findings is if the immunodominance interplay between Gag and Env and potentially other antigens in prime-boost regimens is also playing a role after infection in vaccinated hosts. We have observed vaccine induced changes in post-exposure epitope targeting in inbred animals challenged with lymphocytic choriomeningitis virus [51], but this study is the first to report Env immunodominance over Gag at the whole antigen level in outbred animals. In SIV models, immunodominant responses towards easily mutated epitopes have been found to delay responses towards other epitopes that are harder for the virus to mutate, thus reducing the effect of vaccination [52]. In summary, our study demonstrates that interantigenic immunodominance is a critical parameter in prime-boost regimens. Future optimization of this and related regimens should include both heterologous Env and homologous SIV Gag antigens before nonhuman primate efficacy trials.

## Conclusions

Our study demonstrates the profound impact the choice of Gag can have, when used as a VLP scaffold in prime-boost regimens, on both antibody responses and T cell responses. The broad and increased T cell responses induced by homologous Gag immunization also implicate that these widely sought after responses can be induced by full length protein in most animals, while obtaining humoral responses against Env. Future studies should include analysis of Gag and CE responses to more distant Gag sequences, to provide an even broader knowledge of the perspective of homologous virus encoded VLP vaccines.

## Abbreviations

Env: envelope
VLP: virus-like particle
HIV: human immunodeficiency virus
CE: conserved elements
MVA: modified vaccinia ankara
Ad: adenovirus
CON B: clade B consensus
mAb: monoclonal antibody
i.m.: intramuscularly
ConA: concanavalin A
AdSgSe: huAd5 encoding SIVmac239 *env* with a SIVmac239 *gag* sequence
AdHgSe: huAd5 encoding SIVmac239 *env* with a HIV-1 CON B *gag* sequence
MVAgpe: MVA vector encoding SIVmac239 *gag*, *pol* and *env*
PFU: plaque forming units
PBS: phosphate buffered saline
HRP: horseradish peroxidase
